# Typhoid and Paratyphoid Cost of Illness in Nepal: Patient and Health Facility Costs From the Surveillance for Enteric Fever in Asia Project II

**DOI:** 10.1093/cid/ciaa1335

**Published:** 2020-12-01

**Authors:** Nelly Mejia, Taiwo Abimbola, Jason R Andrews, Krista Vaidya, Dipesh Tamrakar, Sailesh Pradhan, Rajani Shakya, Denise O Garrett, Kashmira Date, Sarah W Pallas

**Affiliations:** 1 Global Immunization Division, US Centers for Disease Control and Prevention, Atlanta, Georgia, USA; 2 Stanford University, Palo Alto, California, USA; 3 Dhulikhel Hospital, Kathmandu University Hospital, Dhulikhel, Nepal; 4 Kathmandu University School of Medical Sciences, Dhulikhel, Nepal; 5 Kathmandu Medical College and Teaching Hospital, Kathmandu, Nepal; 6 Applied Epidemiology, Sabin Vaccine Institute, Washington, DC, USA

**Keywords:** typhoid, paratyphoid, enteric fever, cost of illness, Nepal

## Abstract

**Background:**

Enteric fever is endemic in Nepal and its economic burden is unknown. The objective of this study was to estimate the cost of illness due to enteric fever (typhoid and paratyphoid) at selected sites in Nepal.

**Methods:**

We implemented a study at 2 hospitals in Nepal to estimate the cost per case of enteric fever from the perspectives of patients, caregivers, and healthcare providers. We collected direct medical, nonmedical, and indirect costs per blood culture–confirmed case incurred by patients and their caregivers from illness onset until after enrollment and 6 weeks later. We estimated healthcare provider direct medical economic costs based on quantities and prices of resources used to diagnose and treat enteric fever, and procedure frequencies received at these facilities by enrolled patients. We collected costs in Nepalese rupees and converted them into 2018 US dollars.

**Results:**

We collected patient and caregiver cost of illness information for 395 patients, with a median cost of illness per case of $59.99 (IQR, $24.04–$151.23). Median direct medical and nonmedical costs per case represented ~3.5% of annual individual labor income. From the healthcare provider perspective, the average direct medical economic cost per case was $79.80 (range, $71.54 [hospital B], $93.43 [hospital A]).

**Conclusions:**

Enteric fever can impose a considerable economic burden on patients, caregivers, and health facilities in Nepal. These new estimates of enteric fever cost of illness can improve evaluation and modeling of the costs and benefits of enteric fever–prevention measures.

Each year, there are an estimated 10.9 million cases of typhoid fever and 3.4 million cases of paratyphoid fever globally [1], mostly in tropical low- and middle-income countries with populations that lack adequate access to safe water, sanitation, and hygiene. The economic burden of typhoid and paratyphoid fevers (ie, enteric fever) includes (as for any other disease) direct costs of medical care as well as indirect costs of lost productivity due to illness (and in severe cases, death), which are borne by households, the health system, government, and the broader economy and society [2]. Antibiotic-resistant strains of typhoid and paratyphoid, which are increasingly observed in multiple settings around the world [3], also increase the economic burden of enteric fever as more expensive drugs must be used for treatment.

Enteric fever is endemic in Nepal, a low-income country with a population of 28 million [4]. There has been 1 previous study of the cost of illness (COI) due to typhoid fever in Nepal [5]. However, that study did not consider costs from the healthcare provider perspective, and included only a small, purposively selected sample (n = 20 patients with blood culture diagnosis of typhoid within the previous 6 months) with costs collected through qualitative interview. Studies of typhoid COI were also conducted in other Asian countries (India, Pakistan, Indonesia, Vietnam, China), but with data collected over 15 years ago [5–7] these studies might no longer reflect patterns of healthcare and price levels, given the rapid economic development in the region over this period. Except for the studies in Bangladesh and Pakistan in 2018 from the Surveillance for Enteric Fever in Asia Project (SEAP) II also included in this Supplement [8, 9], previous studies focused on typhoid rather than paratyphoid and lacked detail on specific diagnostic and treatment procedures for enteric fever.

Estimates of the economic burden of enteric fever in endemic countries, such as Nepal, are critical inputs to evaluations of investments in typhoid vaccination—including new typhoid Vi-conjugate vaccines recently recommended and prequalified by the World Health Organization (WHO) and introduced in Pakistan in 2019—and other public health strategies to eliminate enteric fever such as increased access to improved water, sanitation, and hygiene [3, 10].

To help fill this evidence gap, this study presents detailed procedure-level COI estimates for both typhoid and paratyphoid in Nepal based on data collection from a large patient sample recruited through a prospective enteric fever surveillance study (SEAP II) from 2 perspectives: (1) patient and caregiver and (2) healthcare provider.

## METHODS

### Study Setting

This COI study was conducted as a component of SEAP II at 2 teaching hospitals, one in an urban setting in Kathmandu and the other in a peri-urban setting approximately 30 km from Kathmandu. These health facilities were selected based on their laboratory capacity to perform blood culture testing for typhoid and paratyphoid, and they were not intended to be representative of health facilities at any geographical level in Nepal.

### Cost of Illness From the Patient and Caregiver Perspective

#### Study Design

Patients eligible for enrollment in the COI component were those enrolled in SEAP II through the 2 hospitals between September 2016 and December 2018 who were blood culture–confirmed cases of *Salmonella* (*S*.) Typhi or Paratyphi, or with a nontraumatic terminal ileal perforation regardless of blood culture result. Patient and caregiver COI was defined to include (1) direct medical costs (ie, monetary value of health facility registration fees, clinical examination, inpatient stay, laboratory tests, drugs and medications, and other diagnostic and treatment services [eg, X-ray, surgery]), (2) direct nonmedical costs (ie, monetary value of transport, food, and lodging and care services for family members), and (3) indirect costs of lost school and work time due to the episode of enteric fever. Indirect costs were only monetized for patients and caregivers aged 18 years or older, valued at the mean of the self-reported wage ranges (eg, if respondents reported monthly salary in the range of 0–1200 Nepalese rupees, the midpoint of this range—600 rupees—was used to value their time); school days missed were not monetized. Indirect costs included paid workdays lost and sick-leave days used for the patient to seek/receive care and for the caregiver(s) to accompany/provide care. The value of the time spent by caregivers who did not routinely earn a wage (eg, unpaid household labor) only included the time spent at health facilities and was not monetized. Costs from any funding source (except from the healthcare provider) were included. Excluded were the costs of drugs, diagnostics, and therapies not related to enteric fever (eg, antimalarial medications, comorbidities and chronic conditions), any services that patients received at no charge, and intangible costs of pain and suffering.

#### Data Collection

Cost questionnaires were originally developed in English, piloted in the sites, and then translated into Nepali. Questionnaires were administered in Nepali by the same bilingual interviewers as for the surveillance component of SEAP II, with data recorded electronically via tablet. Cost data were collected from patients (if ≥16 years) or their caregivers by phone at 2 time points: (1) 2 to 3 days after a blood culture result or hospital discharge, whichever came later, and (2) 6 weeks (~42 days) after study enrollment (at the same time as the surveillance component follow-up call). The first questionnaire collected data on costs incurred from illness onset through the patient’s return home after the enrollment visit, and the second questionnaire collected costs incurred after the enrollment visit until the follow-up call.

#### Cost of Illness Measures and Data Analysis

The COI measures included median direct medical and nonmedical costs (by subcategory and overall), median number of days of school lost by patients, median number of days of work lost and sick leave used by patients and/or caregivers, the median wages lost by the patient and/or caregiver, and median total COI during the episode of enteric fever. Total COI from the patient and caregiver perspective due to an episode of enteric fever was calculated as the sum of the direct medical costs, direct nonmedical costs, and indirect costs. Median and the interquartile range of COI for each measure were calculated for enteric fever (typhoid and paratyphoid cases and nontraumatic terminal ileal perforation cases combined), and separately for typhoid and paratyphoid in sensitivity analyses.

Costs were collected in Nepalese rupees, adjusted to 2018 values based on inflation rates [4], and converted into 2018 US dollars using the annual average exchange rate for 2018 (109.68 rupees per US dollar [11]). Missing wage information for patients or caregivers was imputed with the median wage of the respondent sample.

Sensitivity analyses were conducted without outliers for each category of patient and caregiver costs. Outliers were defined as the observations with values above or below 2.24 standard deviations from the mean [12]. Sensitivity analyses were also conducted to estimate productivity losses of patients and caregivers who did not routinely earn a wage under 2 wage assumptions: (1) the median wage reported by the respondent sample and (2) the minimum daily wage rate of 517 rupees (or US $4.71 [13]). Analyses were also conducted separated for patients with *S.* Typhi and *S.* Paratyphi.

### Cost of Illness From the Healthcare Provider Perspective

#### Study Design

The COI from the health care provider perspective was estimated as the direct medical economic costs (ie, the value of all resources used, not only financial outlays) to the health facility to diagnose and treat a patient with enteric fever and its associated complications. The health facility sample comprised the same 2 SEAP II hospitals as in the surveillance and patient and caregiver COI components.

Activity-based macro-costing was used to estimate the cost of procedures for which resource use per unit was assumed not to differ between patients with enteric fever and patients with other diseases (excluding drug costs)—namely, outpatient visits, inpatient bed days, emergency visits, intensive care unit bed days, outpatient surgery visits, and inpatient surgery bed days, as well as to allocate cross-cutting administrative and clinical supportive services (eg, laundry and waste disposal). Ingredients-based micro-costing was used to estimate the costs of specific procedures for which activity-based macro-costing by ward could not be conducted because resource use per unit varied by procedure—namely, blood draws, blood culture tests, complete blood count (CBC) tests, abdominal X-rays, abdominal ultrasounds, surgeries for intestinal perforation, and gallbladder surgeries.

Resource inputs included in the cost estimation were personnel time and salaries, materials and supplies, equipment and instruments, contracted services, equivalent rental value of the building space, administrative services, and clinical support services (eg, laundry, cleaning, patient meals). Excluded costs were those associated with magnetic resonance imaging and computed tomography scans (which were reportedly infrequently used for enteric fever diagnosis in the study sites); patient registration, Widal test, C-reactive protein test, medication costs paid for by patients (rather than by the hospital as part of procedure provision), costs unrelated to enteric fever (eg, antimalarial treatment, comorbidities, chronic conditions), nonclinical costs (eg, teaching salaries and classroom space) for the medical school aspects of these teaching hospital sites, evaluation-specific costs, and value of study team staff time for project management, technical assistance, and evaluation.

#### Data Collection

Data were collected using a Microsoft Excel tool that had been piloted in the sites. Prices and quantities of resources used, as well as service volumes, for the 2015–2016 fiscal year were collected using paper-based versions of the tool in local currency (Nepali rupees), and then entered into electronic versions of the tool. Data were collected during February–June 2017 from annual financial reports, administrative records, on-site observation, and interviews with administrative and medical staff by local SEAP II study team staff, with technical assistance from US Centers for Disease Control and Prevention (CDC) staff. Missing prices of supplies/materials or equipment/instruments were imputed with data from the other health facility or, if unavailable from either health facility, the United Nations Children’s Fund (UNICEF) supply catalog [14]. Data on the frequencies of procedures conducted for the patients with blood culture–confirmed enteric fever or nontraumatic ileal perforation in these health facilities were collected through the SEAP II surveillance component during September 2016–December 2018.

#### Cost of Illness Measures and Data Analysis

Data were analyzed in Microsoft Excel. For procedure costs estimated using ingredients-based micro-costing, the unit cost per clinical procedure was calculated as the sum of the products of the quantity of resources used in that procedure multiplied by that resource’s price (or monetary value), as follows:

$$=\underset{j=1}{\sum^{N}}(quantity\;of\;resource\;input\;used_{ij}\text{*}price\;of\;resource\;input_{j})$$

where *i* is the procedure and *j* indexes each resource input used in the procedure up to *N* resources.

For procedure costs estimated using activity-based costing, the monetary value of all resources (eg, equipment, supplies) used in that service ward over the previous year was calculated as the sum of the products of the quantity of resources used in that ward multiplied by that resource’s price (or monetary value) and then divided by that ward’s procedure volume, as follows:

$$=\;\underset{j=1}{\sum^{N}}(quantity\;of\;resource\;input\;used_{wj}\text{*}price\;of\;resource\;input_{j})/{\left(service\;volume\right)}_{w}$$

where *w* is the ward and *j* indexes each resource input used in the ward up to *N* resources.

To each of these types of procedure costs was added a fraction of the hospital-level cross-cutting administrative and clinical supportive services, which were allocated evenly across all services in the hospital. Utilities and cross-cutting clinical supportive services were excluded in hospital B due to missing information.

The average direct economic medical cost per case of enteric fever was calculated by multiplying the unit cost per clinical procedure by the procedure’s frequency in the patient cohort of enteric fever cases identified through blood culture confirmation or nontraumatic ileal perforation from the surveillance study component; these costs were summed across all procedures then divided by the number of enteric fever cases from the surveillance component, as follows:

$$=\frac{\sum_{k=1}^{N}(health\;facility\;unit\;cost\;procedure_{i}\text{*}frequency\;of\;procedure_{ik})}{Total\;number\;of\;confirmed\;enteric\;fever\;cases\;(N)}$$

where *i* is the procedure, *k* indexes the confirmed enteric fever cases at the health facility, and *N* is the total number of enteric fever cases identified at the health facility during the surveillance study period.

Health facility costs had the same inflation and exchange rate adjustments than the patient and caregiver COI costs.

### Ethical Considerations

The study protocol was approved by the Nepal Health Research Council Ethical Review Board Approval and the Stanford University Institutional Review Board. In accordance with the human subjects review procedures of the US CDC, it was determined that the CDC was not formally engaged in human subjects research.

## RESULTS

### Cost of Illness From the Patient and Caregiver Perspective

#### Patient Characteristics

There were 395 patients who (directly or through a caregiver) responded to the first cost questionnaire covering costs through the enrollment visit, of whom 364 (92.2%) responded to the second cost questionnaire covering costs up to the 6-week follow-up call (Table 1). Of this 395-patient sample, 1.3% were younger than 2 years old, 2.5% were 2 to 5 years old, 33.9% were 5 to 17 years old, and 62.3% were aged 18 years or older; 59.5% of patients were male. All included cases were blood culture–positive for either *S.* Typhi (85.6%) or *S.* Paratyphi (15.4%). Most patients’ households had a mobile phone (94.4%), electricity (94.4%), a household flush toilet (91.9%), and a cement roof (77.7%); and 36.7% reported treating their drinking water by boiling or other methods.

**Table 1.** Patient and Caregiver Cost of Illness Due to Enteric Fever: Sample Characteristics—Nepal, September 2016–December 2018

Characteristics	n	%
Respondents		
Patients responding to enrollment cost questionnaire	395	100.0
Patients responding to 6-week follow-up cost questionnaire	364	92.2
Patients who died of enteric fever	0	0.0
Age group		
<2	5	1.3
2–4 years	10	2.5
5–17 years	134	33.9
≥18 years	246	62.3
Sex		
Male	160	40.5
Female	235	59.5
Blood culture result		
*Salmonella* Typhi positive	338	85.6
*Salmonella* Paratyphi positive	57	14.4
Not positive for either *Salmonella* Typhi or Paratyphi (surgical cases)	0	0.0
Household with mobile phone		
Yes	373	94.4
No	3	0.8
Did not respond	19	4.8
Households with electricity		
Yes	373	94.4
No	3	0.8
Did not respond	19	4.8
Households with car/motorcycle		
Yes	191	48.4
No	185	46.8
Did not respond	19	4.8
Household roof material		
Cement	307	77.7
Metal sheets, mats, ceramic, shingles	85	21.5
Natural materials	3	0.8
Households with sanitation		
Household flush to sewer system, septic tank, somewhere else	363	91.9
Household pit latrine, bucket or hanging toilet, communal toilet, other	13	3.3
Did not respond	19	4.8
Drinking water treated at home		
Boil	63	15.9
Chlorine liquid, powder, or tablets	72	18.2
Other	10	2.5
Do not treat water	148	37.5
Did not respond	101	25.6

#### Patient and Caregiver Direct Medical and Nonmedical Costs

**Figure 1.**
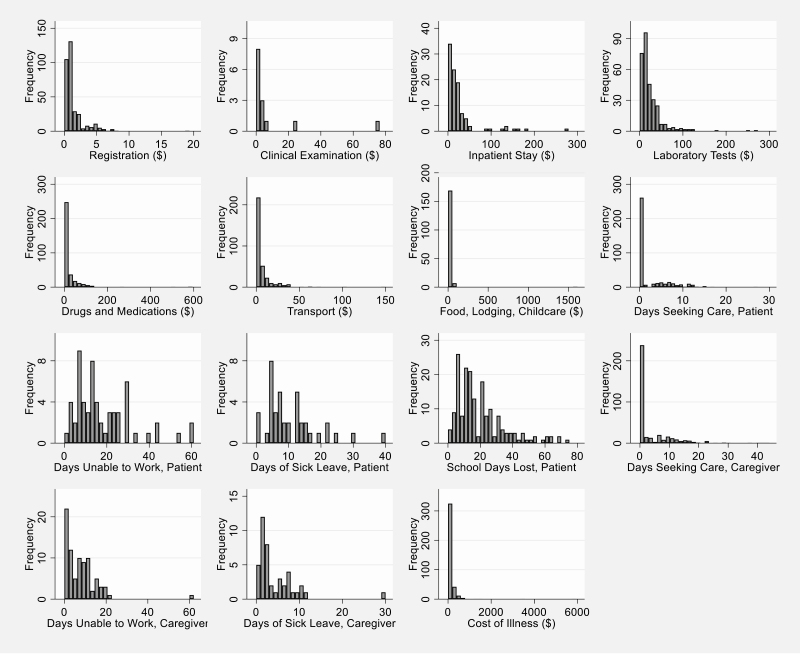
Distribution of cost of illness elements from the patient and caregiver perspective, Nepal, September 2016–December 2018 (N = 395 patients; 2018 US dollars).

Of the 395 patients with blood culture–confirmed enteric fever, 377 (95.4%) reported direct medical costs (Table 2). Median direct medical costs were US $34.25 (interquartile range [IQR], US $18.24–$91.17). Direct medical expenses were the largest component of the COI for all patients, as well as for patients who reported inpatient care and patients who only reported outpatient care. The costs reported by patients who received inpatient care were higher than those for patients only receiving outpatient care in most cost subcategories. Although not all respondents were able to recall the costs they had paid for specific procedures, the most frequently reported costs were drugs and medications (reported by 89.7%; median cost: US $9.39), registration (reported by 88.3%; median cost: US $0.76), and laboratory tests (reported by 78.2%; median cost: US $16.41) (Figure 1).

**Table 2.** Patient and Caregiver Cost of Illness Due to Enteric Fever: Direct Medical, Direct Nonmedical, Indirect, and Total Costs—Nepal, September 2016–December 2018

	Patients Who Did Not Report Any Inpatient Care Expenses^a^				Patients Who Reported Inpatient Care Expenses^a^				All Patients			
Cost Type	n	Median	25th Pctl	75th Pctl	n	Median	25th Pctl	75th Pctl	n	Median	25th Pctl	75th Pctl
Direct medical costs, 2018 US $												
Total direct medical costs	277	24.86	14.59	45.59	100	139.38	95.73	216.54	377	34.25	18.24	91.17
Registration	250	0.73	0.64	1.33	83	1.09	0.64	2.46	333	0.76	0.64	1.66
Clinical examination	10	1.54	0.39	3.28	4	2.57	1.14	39.82	14	1.86	0.46	3.32
Inpatient stay	0	N/A	N/A	N/A	100	12.79	7.29	26.67	100	12.79	7.29	26.67
Laboratory tests	227	13.68	6.38	20.70	82	37.23	18.23	56.93	309	16.41	9.35	29.18
Drugs and medications	252	7.84	4.43	11.72	86	47.43	19.15	83.52	338	9.39	4.92	21.88
Other services^b^	2	8.25	3.68	12.81	5	3.32	3.32	3.61	7	3.61	3.32	12.81
Direct nonmedical costs, 2018 US $												
Total direct nonmedical costs	258	3.21	1.37	7.59	96	16.37	4.97	48.83	354	4.56	1.71	13.73
Transport	246	2.05	0.95	5.47	92	6.22	2.74	17.10	338	2.74	1.09	7.97
Food, lodging, child care	123	2.28	1.37	4.90	54	27.35	13.68	41.32	177	4.56	1.73	18.23
Direct medical and nonmedical expenses, 2018 US $												
Direct medical and nonmedical pre-enrollment expense	192	2.51	0.71	9.63	77	6.38	3.19	18.98	269	3.65	0.91	12.31
Total direct medical and nonmedical expenses	286	27.95	17.16	50.15	100	168.22	108.79	268.83	386	39.57	20.21	104.37
Indirect costs—patient												
Days spent seeking care	282	0.21	0.13	0.38	100	7.00	4.00	10.00	382	0.29	0.13	4.00
Days unable to work	51	14.00	8.00	26.00	9	20.00	14.00	24.00	60	14.00	8.00	25.50
Days of sick leave	35	7.00	5.00	13.00	5	19.00	12.00	25.00	40	7.50	5.00	14.10
School days lost	117	14.00	7.00	22.00	63	21.00	12.00	33.00	180	14.50	9.00	26.00
Total productivity loss, 2018 US $	76	44.10	26.33	84.63	12	73.35	49.82	161.04	88	49.24	26.87	92.15
Indirect costs—caregiver												
Days spent accompanying the patient seeking care	270	0.29	0.17	1.00	100	10.00	6.00	14.40	370	1.00	0.19	6.29
Days unable to work	44	3.00	1.00	7.00	39	10.00	6.00	14.00	83	7.00	2.00	11.00
Days of sick leave	31	2.00	1.00	3.00	12	6.50	4.50	9.50	43	2.00	1.00	6.00
Total productivity loss, 2018 US $	72	7.83	3.76	22.57	48	33.85	22.57	50.78	120	16.43	7.52	37.61
Cost of illness, 2018 US $												
Total cost of illness per case	288	38.90	19.9	77.19	100	189.53	144.56	317.98	388	59.99	24.04	151.23
Total cost of illness per case without the value of sick leave	286	32.96	18.5	68.41	100	189.53	142.41	292.52	386	50.92	21.88	146.03

N = 395.

Abbreviations: N/A, not applicable because it excludes inpatient care; 25th Pctl, 25th percentile; 75th Pctl, 75th percentile.

^a^Inpatient care expenses: expenses for inpatient care regardless of patient recruitment location (outpatient care, inpatient care, hospital laboratory, surgery).

^b^Other services that patients did not report in the above categories (eg, medical materials or equipment for surgery).

Direct nonmedical costs, such as transport, food, lodging, and child care, were reported by 354 patients (93.9%), with a median cost of US $4.56 (IQR, US $1.71–$13.73) (Table 2). Transport was the most frequently reported cost of this type (reported by 95.5% of those with any direct nonmedical costs; median cost: US $2.74) (Figure 1). Median direct nonmedical costs (overall and by subcategories) were higher for patients reporting inpatient care costs compared with patients reporting only outpatient care costs. Median direct medical and nonmedical costs per case represented approximately 3.5% of annual individual labor income for all patients compared with annualized median wage rates reported in the sample (US $1083.26).

Out of 395 patients, 269 (68.1%) reported direct expenses for seeking care at health facilities before enrollment in the study (Table 2), half of whom (133) sought care at pharmacies. Median direct medical and nonmedical costs incurred before enrollment were US $3.65 (IQR, US $0.91–$12.31), which represented 9.2% of the median total direct medical and nonmedical costs for all patients.

#### Patient and Caregiver Indirect Costs

From illness onset until the time of COI interview at 6 weeks (or up to the first questionnaire if the second questionnaire was not answered), a median of 0.29 days were spent by 382 patients seeking and receiving care (IQR, 0.13–4.00 days) (Table 2, Figure 1). A median of 14.50 days of school were lost (IQR, 9.00–26.00 days) by 180 patients. There were 60 patients reporting a median of 14.00 lost workdays (IQR, 8.00–25.50 days) and 40 patients reporting a median of 7.50 sick-leave days used (IQR, 5.00–14.10 days). On average, patients received care from 2.0 caregivers. Caregivers for 83 patients were unable to work for a median of 7.00 days (IQR, 2.00–11.00 days); in addition, caregivers for 43 patients used a median of 2.00 days of sick leave (IQR, 1.00–6.00 days). When valued at the median of patients’ and caregivers’ reported wage rate ranges, these days of work lost plus sick leave used equate to a median productivity loss of US $49.24 (IQR, US $26.87–$92.15) for patients and US $16.43 (IQR, US $7.52–37.61) for caregivers. Median productivity losses were greater for hospitalized patients in all subcategories of indirect costs.

#### Median Cost per Case of Enteric Fever

After adding direct medical costs, direct nonmedical costs, and indirect costs of patient and caregiver productivity losses, the median cost of illness per case of enteric fever from the patient and caregiver perspective was US $59.99 (IQR, US $24.04 –$151.23) for all patients (Table 2, Figure 1). Similarly to the trend observed by cost categories, the median COI for patient receiving inpatient care was higher than the COI of patients only reporting outpatient care (4.9 times higher). After removing the value of the sick leave (potentially borne by a third party and not by the patient and caregivers), the median COI decreased to US $50.92 (IQR, US $21.88 –$146.03).

#### Sensitivity Analysis

Results changed minimally as expected when removing outlier values (slight change in median COI from US $59.99 to US $56.35), imputing wages for unpaid labor (higher median COI of US $66.00 for all patients), and using the national minimum wage rates for unpaid labor of caregivers and patients (higher median COI of US $67.32 for all patients) (Tables 3 and 4). Results were qualitatively similar for typhoid (85.6%) and paratyphoid (14.4%) cases, with higher median costs for paratyphoid cases in some subcategories but lower median COI overall (US $51.95) compared with typhoid cases (US $59.86) for all patients (Tables 5 and 6).

**Table 3.** Sensitivity Analysis of Patient and Caregiver Cost of Illness Due to Enteric Fever: Direct Medical, Direct Nonmedical, Indirect, and Total Costs When Excluding Outliers—Nepal, September 2016–December 2018

	Patients Who Did Not Report Any Inpatient Care Expenses^a^				Patients Who Reported Inpatient Care Expenses^a^				All Patients			
Cost Type	n	Median	25th Pctl	75th Pctl	n	Median	25th Pctl	75th Pctl	n	Median	25th Pctl	75th Pctl
Direct medical costs, 2018 US $												
Total direct medical costs	275	24.86	14.59	43.54	98	138.45	95.73	204.00	373	34.19	18.23	91.17
Registration	247	0.73	0.64	1.28	75	1.09	0.64	1.82	322	0.73	0.64	1.37
Clinical examination	10	1.54	0.39	3.28	3	1.82	0.46	3.32	13	1.82	0.46	3.28
Inpatient stay	0	N/A	N/A	N/A	93	12.34	7.29	23.71	93	12.34	7.29	23.71
Laboratory tests	227	13.68	6.38	20.70	71	31.17	18.04	42.85	298	15.33	8.94	27.44
Drugs and medications	251	7.84	4.31	11.67	83	43.76	18.23	75.91	334	9.30	4.89	21.43
Other services^b^	2	8.25	3.68	12.81	5	3.32	3.32	3.61	7	3.61	3.32	12.81
Direct nonmedical costs, 2018 US $												
Total direct nonmedical costs	257	3.19	1.37	7.48	96	16.37	4.97	48.83	353	4.56	1.71	13.68
Transport	244	1.96	0.95	5.29	87	5.84	2.28	14.23	331	2.55	1.09	7.29
Food, lodging, child care	122	2.28	1.37	4.74	55	27.35	13.68	41.32	176	4.56	1.66	18.23
Indirect costs—patient												
Days spent seeking care	278	0.21	0.13	0.38	89	6.00	4.00	8.21	367	0.27	0.13	3.08
Days unable to work	48	14.00	7.50	22.50	9	20.00	14.00	24.00	57	14.00	8.00	24.00
Days of sick leave	34	7.00	5.00	13.00	4	15.50	6.10	22.00	38	7.00	5.00	13.00
School days lost	113	14.00	7.00	21.00	59	16.00	12.00	31.00	172	14.00	8.50	24.00
Total productivity loss, 2018 US $	74	43.01	26.33	82.75	11	71.47	46.97	156.58	85	46.97	26.33	82.75
Indirect costs—caregiver												
Days spent accompanying the patient seeking care	267	0.29	0.17	1.00	88	9.06	5.61	12.13	355	0.63	0.17	5.00
Days unable to work	43	3.00	1.00	7.00	39	10.00	6.00	14.00	82	7.00	2.00	11.00
Days of sick leave	30	1.50	1.00	2.00	12	6.50	4.50	9.50	42	2.00	1.00	6.00
Total productivity loss, 2018 US $	70	7.83	3.76	18.81	48	33.85	22.57	50.78	118	15.66	7.52	37.61
Cost of illness, 2018 US $												
Total cost of illness per case	286	38.06	19.88	76.22	98	188.19	144.42	309.29	384	56.35	23.80	146.48

Abbreviations: N/A, not applicable because it excludes inpatient care; 25th Pctl, 25th percentile; 75th Pctl, 75th percentile.

^a^Inpatient care expenses: expenses for inpatient care regardless of patient recruitment location (outpatient care, inpatient care, hospital laboratory, surgery).

^b^Other services that patients did not report in the above categories (eg, medical materials or equipment for surgery).

**Table 4.** Sensitivity Analysis of Patient and Caregiver Cost of Illness Due to Enteric Fever: Indirect and Total Costs When Using Alternative Wage Rates and Including Unpaid Labor—Nepal, September 2016–December 2018

	Patients Who Did Not Report Any Inpatient Care Expenses^a^				Patients Who Reported Inpatient Care Expenses^a^				All Patients			
Cost Type	n	Median	25th Pctl	75th Pctl	n	Median	25th Pctl	75th Pctl	n	Median	25th Pctl	75th Pctl
Indirect costs—imputing median wage in the sample for unpaid patient and caregivers’ time												
Total productivity loss	289	5.02	1.28	32.64	100	80.54	46.53	127.47	389	16.93	1.83	68.33
Cost of illness—imputing median wage in the sample for unpaid patient and caregivers’ time												
Total cost of illness per case	292	43.89	21.64	84.43	100	255.71	185.62	387.21	392	66.00	26.25	187.30
Indirect costs—imputing country minimum wage for unpaid patient and caregivers’ time												
Total productivity loss	289	6.19	1.59	34.38	100	95.13	57.94	154.69	389	19.31	2.36	82.25
Cost of illness—imputing country minimum wage for unpaid patient and caregivers’ time												
Total cost of illness per case	292	44.69	22.67	85.52	100	270.94	203.33	406.65	392	67.32	26.66	196.13

Data are presented in 2018 US dollars. N = 395.

Abbreviations: 25th Pctl, 25th percentile; 75th Pctl, 75th percentile.

^a^Inpatient care expenses: expenses for inpatient care regardless of patient recruitment location (outpatient care, inpatient care, hospital laboratory, surgery).

**Table 5.** Patient and Caregiver Cost of Illness Due to Typhoid Fever: Direct Medical, Direct Nonmedical, Indirect, and Total Costs—Nepal, September 2016–December 2018

	Patients Who Did Not Report Any Inpatient Care Expenses^a^				Patients Who Reported Inpatient Care Expenses^a^				All Patients			
Cost Type	n	Median	25th Pctl	75th Pctl	n	Median	25th Pctl	75th Pctl	n	Median	25th Pctl	75th Pctl
Direct medical costs, 2018 US $												
Total direct medical costs	234	26.79	15.73	46.09	90	139.38	95.73	227.94	324	36.70	19.56	96.19
Registration	211	0.73	0.64	1.37	77	1.19	0.68	2.46	288	0.82	0.64	1.82
Clinical examination	8	1.54	0.43	3.95	4	2.57	1.14	39.82	12	1.86	0.47	4.27
Inpatient stay	0	N/A	N/A	N/A	90	12.76	7.29	28.72	90	12.76	7.29	28.72
Laboratory tests	191	13.79	7.29	22.25	73	37.95	18.23	56.53	264	16.41	10.21	30.11
Drugs and medications	209	7.98	4.56	11.94	78	47.49	21.43	83.52	287	9.57	5.01	24.67
Other services^b^	2	8.25	3.68	12.81	5	3.32	3.32	3.61	7	3.61	3.32	12.81
Direct nonmedical costs, 2018 US $												
Total direct nonmedical costs	218	3.46	1.37	8.54	87	16.50	5.47	50.15	305	4.74	1.82	14.42
Transport	208	2.19	1.09	5.47	84	7.07	3.72	17.10	292	2.92	1.19	9.03
Food, lodging, child care	98	2.37	1.37	5.47	50	27.35	13.68	41.32	148	5.15	1.79	22.79
Indirect costs—patient												
Days spent seeking care	238	0.21	0.13	0.38	90	7.00	4.00	10.00	328	0.31	0.13	4.04
Days unable to work	39	14.00	7.00	25.00	7	20.00	14.00	44.00	46	14.00	8.00	25.00
Days of sick leave	27	7.00	5.00	14.00	3	12.00	0.21	40.00	30	7.00	5.00	14.00
School days lost	102	14.00	7.00	23.00	60	21.00	12.00	33.00	162	15.00	9.00	26.00
Total productivity loss, 2018 US $	58	41.75	26.33	82.75	9	75.23	52.66	156.58	67	46.97	27.40	82.75
Indirect costs—caregiver												
Days spent accompanying the patient seeking care	228	0.29	0.17	1.00	90	10.19	6.13	15.00	318	1.00	0.19	7.02
Days unable to work	36	2.50	1.00	8.00	36	10.00	6.50	13.50	72	7.00	2.00	11.50
Days of sick leave	26	2.00	1.00	3.00	12	6.50	4.50	9.50	38	2.00	1.00	6.00
Total productivity loss, 2018 US $	59	7.83	3.76	26.33	45	33.85	22.57	48.90	104	18.81	7.52	40.91
Cost of illness, 2018 US $												
Total cost of illness per case	242	38.90	20.21	77.34	90	189.60	144.70	309.29	332	59.86	24.74	151.23

N = 338.

Abbreviations: N/A, not applicable because it excludes inpatient care; 25th Pctl, 25th percentile; 75th Pctl, 75th percentile.

^a^Inpatient care expenses: expenses for inpatient care regardless of patient recruitment location (outpatient care, inpatient care, hospital laboratory, surgery).

^b^Other services that patients did not report in the above categories (eg, medical materials or equipment for surgery).

**Table 6.** Patient and Caregiver Cost of Illness Due to Paratyphoid Fever: Direct Medical, Direct Nonmedical, Indirect, and Total Costs—Nepal, September 2016–December 2018

	Patients Who Did Not Report Any Inpatient Care Expenses^a^				Patients Who Reported Inpatient Care Expenses^a^				All Patients			
Cost Type	n	Median	25th Pctl	75th Pctl	n	Median	25th Pctl	75th Pctl	n	Median	25th Pctl	75th Pctl
Direct medical costs, 2018 US $												
Total direct medical costs	43	20.06	12.05	32.09	10	141.00	56.05	205.14	53	27.57	12.81	29.63
Registration	39	0.73	0.64	1.23	6	0.91	0.64	1.09	45	0.73	0.64	1.14
Clinical examination	2	1.83	0.38	3.28	0	…	…	…	2	1.83	0.38	3.28
Inpatient stay	0	N/A	N/A	N/A	10	15.89	4.74	25.53	10	15.89	4.74	25.53
Laboratory tests	36	9.23	4.63	18.48	9	31.17	19.88	71.16	45	12.26	5.33	23.72
Drugs and medications	43	7.59	3.80	11.12	8	37.84	6.18	103.59	51	7.94	4.24	12.76
Other services^b^	0	…	…	…	0	…	…	…	0	…	…	…
Direct nonmedical costs, 2018 US $												
Total direct nonmedical costs	40	2.53	1.26	4.79	9	3.61	0.64	48.32	49	2.64	1.09	5.29
Transport	38	1.47	0.55	2.92	8	2.28	0.60	12.11	46	1.47	0.57	3.61
Food, lodging, child care	25	1.90	0.47	3.42	4	32.85	28.29	46.73	29	2.74	1.09	4.27
Indirect costs—patient												
Days spent seeking care	44	0.21	0.16	0.37	10	5.00	4.00	7.00	54	0.25	0.17	3.33
Days unable to work	12	19.00	11.50	35.00	2	15.50	7.00	24.00	14	19.00	9.00	30.00
Days of sick leave	8	6.50	4.00	13.00	2	22.00	19.00	25.00	10	10.50	4.00	16.00
School days lost	15	14.00	6.00	19.00	3	20.00	12.00	63.00	18	14.00	6.00	20.00
Total productivity loss, 2018 US $	18	59.60	18.81	99.75	3	71.47	26.33	184.30	21	66.54	26.33	99.75
Indirect costs—caregiver												
Days spent accompanying the patient seeking care	42	0.30	0.17	1.00	10	6.05	4.00	10.00	52	0.81	0.17	3.00
Days unable to work	8	3.00	2.00	6.00	3	10.00	4.00	21.00	11	4.00	3.00	10.00
Days of sick leave	5	1.00	1.00	2.00	0	…	…	…	5	1.00	1.00	2.00
Total productivity loss, 2018 US $	13	11.28	3.76	17.20	3	37.61	15.05	78.99	16	11.51	3.76	28.21
Cost of illness, 2018 US $												
Total cost of illness per case	46	37.04	18.03	76.22	10	284.15	59.65	325.96	56	51.95	20.65	140.01

N = 57.

Abbreviations: N/A, not applicable because it excludes inpatient care; 25th Pctl, 25th percentile; 75th Pctl, 75th percentile.

^a^Inpatient care expenses: expenses for inpatient care regardless of patient recruitment location (outpatient care, inpatient care, hospital laboratory, surgery).

^b^Other services that patients did not report in the above categories (eg, medical materials or equipment for surgery).

### Cost of Illness From the Health Provider Perspective

Procedure costs varied across the 2 hospital sites due to differences in service volumes, resource quantities used per procedure, and resource prices. The most costly procedures at both hospitals were intestinal perforation surgery (hospital A: US $280.08; hospital B: US $279.61) and gallbladder surgery (hospital A: US $193.49; hospital B: US $189.57), while the least costly were blood draw (hospital A: not available; hospital B: US $2.02), CBC (hospital A: US $7.36; hospital B: US $5.20), and abdominal X-ray (hospital A: US $4.65; hospital B: US $8.83) (Table 7). Data on the frequencies of some specific procedures were not available from the surveillance component. In addition, data on the prices of some medical items in each hospital were not available in the UNICEF supply catalog or from the other hospital, and therefore it was not feasible to accurately estimate the cost of some procedures. The 265 patients included in the frequency calculations were those enrolled at the 2 hospital sites regardless of whether they consented to participate in the patient and caregiver COI component; it did not include the 135 patients recruited into surveillance through hospital laboratory network sites. The frequency-weighted average direct medical cost per case of enteric fever was US $79.80 (hospital A: US $93.43; hospital B: $71.54).

**Table 7.** Healthcare Provider Cost of Illness Due to Enteric Fever: Procedure Unit Costs and Frequencies and Average Cost per Case of Enteric Fever—Nepal, July 2015–June 2016

	Unit Cost in 2018 US $		Frequency		
Procedure	Hospital A	Hospital B	Hospital A	Hospital B
General services not specific to enteric fever				
Outpatient routine service cost (per patient, per visit)	11.40	7.72	51	101
Inpatient hospital cost (per patient, per day)	16.01	15.27	345	364
Surgical outpatient visit (per patient, per visit)	12.54	8.51	1	0
Surgical inpatient (per patient, per day)	19.56	9.76	0	0
ICU (per patient, per day)	… ^a^	14.45	… ^a^	… ^a^
Services specific to enteric fever				
Gallbladder surgery	193.49	189.57	… ^a^	… ^a^
Surgery for intestinal perforation	280.08	279.61	1	1
Blood culture	19.93	21.13	100	165
Abdominal ultrasound	5.59	6.80	14	47
Complete blood count	7.36	5.20	91	145
Abdominal X-ray^b^	4.65	8.83	44	71
Blood draw	… ^a^	2.02	81	146
Total blood culture–confirmed enteric fever or nontraumatic ileal perforation cases			100	165
Weighted average cost per case by enrollment site	93.43	$71.54	…	…
Weighted average cost per case (both sites)	79.80		…	

N = 265.

Abbreviation: ICU, intensive care unit.

^a^Missing information.

^b^Based on number of chest X-rays as a proxy, as number of abdominal X-rays was not collected in the clinical surveillance component.

## Discussion

We found that costs related to enteric fever were considerably higher among our study population in Nepal. In comparing our cost estimates from Nepal with published cost estimates from other countries, our patient and caregiver COI estimates show higher average direct medical and nonmedical costs than any other country besides China, although only slightly higher than the previously published small-scale Nepal study (Table 8) [5–7, 15]. Within our SEAP country studies, the direct medical and nonmedical COI for Nepal was slightly lower when compared with Bangladesh and less than one-third of the estimated direct medical and nonmedical COI for Pakistan [8, 9]. These higher direct medical and nonmedical costs likely reflect differences in the healthcare systems and organizations included in each study, as well as potentially higher healthcare prices today versus when data were collected for most previous studies in 1995–2003. The lower indirect costs in our study are due to differences in how indirect costs were defined and valued; specifically, in our COI estimates, only the productivity losses of patients and caregivers aged 18 years and older are monetized, whereas other studies monetized productivity losses of those younger than 18. Despite the enormous economic burden and high incidence of enteric fever in Nepal (145 454 illnesses in 2017 [1]), the government has not yet applied for Gavi funding or committed to typhoid Vi-conjugate vaccine introduction. Introducing the typhoid Vi-conjugate vaccines among vulnerable groups would result not only in health benefits by preventing morbidity and mortality but also in substantial economic savings for the beneficiaries.

**Table 8.** Average Costs for a Case of Enteric Fever for Children and Adults in Nepal and Other Countries

	SEAP			Other Studies							
	Nepal	Bangladesh [8]^a^	Pakistan [9]	Nepal [5]	India [6]	Tanzania [15]^b^	Vietnam [7]^c^	Chinac [7]	Indonesia [7]	Pakistan [7]^d^	India [7]
All patients											
Direct (medical and nonmedical), $	111.72	…	$383.46	$101.46	$55.11	$30.27	$59.34	$175.78	$88.83	$64.98	$9.82
Indirect (days of work/sick leave/school lost)	14.29	…	24.42	22	…	…	…	…	…	…	…
Indirect cost of patients and caregivers, $	21.02	…	$56.34	$35.29	$48.02	$146.55	$8.99	$45.25	$65.53	$13.00	$8.18
Adults (≥18 years)											
Direct (medical and nonmedical), $	107.25	…	$441.43	…	…	…	…	$226.25	$163.10	…	…
Indirect (days of work/sick leave/school lost)	10.75	…	30.80	…	…	…	…	…	…	…	…
Indirect cost of patients and caregivers, $	28.38	…	$145.35	…	…	$128.28	…	$62.65	$125.23	…	…
Children (<18 years)											
Direct (medical and nonmedical), $	119.11	$123.47	$362.95	…	…	…	$59.34	$99.20	$43.69	$64.98	$8.17
Indirect (days of work/sick leave/school lost)	20.14	13.87	22.17	…	…	…	…	…	…	…	…
Indirect cost of caregivers, $	11.53	$3.14	$24.84	…	…	$172.65	$8.99	$17.40	$33.49	$13.00	$4.90
Study characteristics											
Perspective	Patient and caregiver			Patient and provider	Patient	Societal	Patient and provider				
Type of enteric fever	Typhoid and paratyphoid			Typhoid	Typhoid	Typhoid	Typhoid				
Indirect costs	Only adults (≥18 years)			Working children and adults	Children and adults	Children and adults	Children and adults				

Data are presented in 2018 US dollars. Results from other countries were adjusted by inflating local currencies using local inflation rates and then exchanging to US dollars. The studies in this table have methodological differences that prevent them to be directly comparable. Sources: references 5–9, 15.

^a^Only includes children <18 years and indirect costs correspond exclusively to caregivers.

^b^In this study children are ≤15 years.

^c^In this study children are 5–17 years.

^d^In this study children are 2–15 years.

Median direct medical costs for all patients and caregivers in our sample and for patients reporting inpatient care costs were 70.1% and 285.2%, respectively, of Nepal’s all-source health expenditure per capita of US $48.87 (2016 value in 2018 US dollars [4]). This illustrates the substantial economic burden posed by enteric fever in this setting. Previous literature defined catastrophic health expenditures as COI that exceeds 10% of annual household income [16–18]; by this standard, if the patients or caregivers who reported productivity losses were the only income earners in their households, then the median direct medical and nonmedical costs per case of enteric fever for patients reporting inpatient care costs (15.5% of annual individual labor income) would be considered catastrophic in this study population (household income was not directly measured in this COI study). The average cost per case of enteric fever from the healthcare provider perspective represented 213% of health expenditure per capita. In this study setting, the average direct medical economic cost to the healthcare provider per case of enteric fever was more than twice the direct medical cost to the patient and caregiver, possibly reflecting the availability of subsidized and free care available at the study site hospitals for patients with limited means.

### Limitations

Our results are subject to several limitations. The patient and site samples are not representative of Nepal and only reflect patients who sought care at these tertiary hospitals; we do not know how costs incurred by patients in other settings in Nepal might differ from those in our study. Thus, results of this study are only valid for similar populations. Patient and caregiver COI interviews were conducted by phone instead of in person, which could have affected the response rate. Self-reported patient and caregiver direct and indirect costs may be subject to recall or reporting biases. A control group to account for potential background patient morbidity and healthcare costs was not included. The study does not model the risk of worsening antimicrobial-resistant enteric fever and its associated costs. Some medical supply prices had to be imputed based on third-party sources for the healthcare provider COI (eg, other hospital and UNICEF), which may have increased or decreased the estimated costs compared with the real ones. Some elements of healthcare provider COI (eg, personnel time per procedure) may be subject to recall or reporting biases. Costs were not combined across perspectives due to the limited health facility sample, which did not represent all health facilities visited by patients at which patient and caregiver costs were incurred, and the limited ability of patients and caregivers to recall and report expenses for specific clinical procedures (eg, by specific type of laboratory test) to match these with health facility costs. Thus, the COI from the patients and caregiver perspective includes the cost of multiple health facilities visited by the same patient, while the COI from the health provider includes the cost of 1 health facility treating a patient.

### Conclusions

Estimates of the economic burden of enteric fever are important to evaluate the value of interventions such as new typhoid conjugate vaccines and improvements to water and sanitation in Nepal and elsewhere. Our COI estimates reported here illustrate that the economic burden of typhoid and paratyphoid is considerable to patients, their caregivers, and healthcare providers in Nepal. Future estimation of COI from a more representative site and patient sample, and including costs to other payers such as government, may help characterize the economic burden of enteric fever from the full societal perspective.

## Notes

***Acknowledgments.*** The authors acknowledge the support provided by the Surveillance for Enteric Fever in Asia Project (SEAP) Nepal team, personnel in the SEAP Nepal study sites; other members of the SEAP project team who supported data collection and management, especially Ashley Tate Longley and Caitlin Barkume; and personnel at the International Centre for Diarrhoeal Disease Research, Bangladesh.

***Disclaimer:*** The results and conclusions in this article are those of the authors and do not necessarily represent the official position or policies of the US Centers for Disease Control and Prevention or other partners.

***Financial support.*** This work was supported by the Bill & Melinda Gates Foundation (grant number INV-008335) and the US Centers for Disease Control and Prevention (CDC). K. D., T. A., N. M., and S. P. report a conditional gift agreement between the Sabin Vaccine Institute (primary grantee for the SEAP grant from the Bill & Melinda Gates Foundation) and the CDC Foundation.

***Supplement sponsorship.*** This supplement is sponsored by the Sabin Vaccine Institute and made possible by a grant from the Bill & Melinda Gates Foundation.

***Potential conflicts of interest.*** The authors: No reported conflicts of interests. All authors have submitted the ICMJE Form for Disclosure of Potential Conflicts of Interest.
